# A Monte-Carlo-Based Network Method for Source Positioning in Bioluminescence Tomography

**DOI:** 10.1155/2007/48989

**Published:** 2007-10-18

**Authors:** Zhun Xu, Xiaolei Song, Xiaomeng Zhang, Jing Bai

**Affiliations:** Department of Biomedical Engineering, School of Medicine, Tsinghua University, Beijing 100084, China

## Abstract

We present an approach based on the improved Levenberg Marquardt (LM) algorithm of backpropagation (BP) neural network to estimate the light source position in bioluminescent imaging. For solving the forward problem, the table-based random sampling algorithm (TBRS), a fast Monte Carlo simulation method we developed before, is employed here. Result shows that BP is an effective method to position the light source.

## 1. INTRODUCTION

Recently developed bioluminescence tomography (BLT), with its noninvasive nature, has become a hotspot in invivo optical imaging which can reveal the molecular and cellular activity through determining the distribution of bioluminescent sources [1, 2]. Therefore, it can be applied to the study of much physiological and pathological processes through small animal imaging, such as monitoring tumor growing and drug delivery, evaluating new therapies, and examining protein and gene functions.

There are two basic points in the reconstruction of BLT, one is the accurate modeling and solving of photon propagation through biologic tissues, and the other is the proper inversion strategy. Most existing reconstruction methods in BLT were based on diffusion approximation of radiative transfer equation. And the diffusion approximation, considered as a linear problem, can be solved by using classical inversion methods such as some regularization skills [2, 3], Newton and modified Newton method [3–5], and other strategies like adaptive finite element [5]. However, using diffusion equation to describe the photon transportation has its limitations in some special cases and solving it accurately is also very difficult [6]. Monte Carlo (MC) approach is always employed to simulate the photon propagation for its accuracy and flexibility [7]. But the time-consuming nature made it seldom used in reconstruction field.

Based on traditional MC method, a table-based random sampling (TBRS) method [8] was developed by Xiaomeng Zhang in our lab, which could remarkably accelerate the computation while keeping the accuracy of MC. In this paper with the TBRS algorithm, the method that simulates the photon transportation, an improved LM algorithm of BP neural network, is used to calculate the position of bioluminescent source approximately, since BP neural network is effective in finding the nonlinearity between the inputs and the outputs, and LM algorithm will speed up the process of training.

## 2. METHODOLOGY

### 2.1. Scheme of the TBRS algorithm

**Figure 1:**
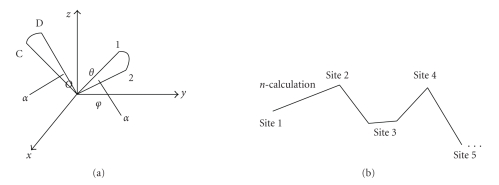
The mapping principle of TBRS is shown in (a). The process of obtaining new sites through n-calculation is described in (b).

The TBRS algorithm is based on a table to find a process in obtaining the positions and directions of scattering photons [8]. This table records a photon's successive N steps of transportation by including the position and direction of the photon during each scattering. For any consecutive n (n≪N) steps in the N steps of photon movement, the TBRS algorithm suggests a possible state of continuous n-step transportation. Thus, TBRS simulation can be started based on the table we have built. With the help of the table, we can randomly take out the continuous n steps from the table and obtain the change of position and direction from one position to the other one within these n steps. Then, it is added to the position and direction of the photon in site 1, the initial position of the photon, through the mapping principal illustrated in Figure 1(a) . Hence, the new position and direction of the photon in site 2 can be obtained.

With the method as mentioned above, the n steps of a photon's transportation are simplified to one step in the simulation. Such approach can be performed again and again to obtain site 3, site 4, and so forth. (refer to Figure 1(b)). Once the photon reaches the inside or outside boundary of the media, it may either be reflected or transmitted, which is determined by Snell's law.

The comparison between TBRS algorithm and conventional Monte Carlo algorithm shows that, with the exact same conditions (size, geometric parameters, and optical parameters of simulation media, computer configuration), the computing time of TBRS is about 40% of conventional Monte Carlo method [8].

### 2.2. The application of backpropagation neural network to the reconstruction

To estimate the bioluminescent source position, we propose an approach based on artificial neural network. It is one of the most active methods in the realm of intelligence control, especially in finding the nonlinearity between the inputs and the outputs even in the absence of enough information about the relationship between them [9].

The first step of the method is to generate proper number of training data, in which the source position is randomly selected and varies in each simulation. TBRS method is employed here to simulate the measurements as it has the accuracy similar to that of Monte Carlo while more timesaving than it. The results obtained from the TBRS are used as the actual measurement data to train the network in the process of reconstruction.

Here we use a single hidden layer of back propagation neural network method where the input layer comprises the actual measurement data, and the output layer is the position of the source, which is in the term of 3 coordinate values.

We consider the training error to be the sum over output units of the squared difference between the desired output $t_{k}$ and the actual output $z_{k}$. So, we can define a criterion function as

(1) $$J(w)=\frac{1}{2}\sum_{k=1}^{3}{\left(t_{k}-z_{k}\right)}^{2},$$

where $\left(t_{1},t_{2},t_{3}\right)$ is the actual coordinate of the source position, the desired output in the process of training, while $\left(z_{1},z_{2},z_{3}\right)$ is the network's output, which can be adjusted each time until J(ω) approaches the given limit.

Once J(ω) drops to a value lower than the error limit through the adjustment of ω, the training ceases and the network is determined. When the actual measurement data is inserted to the input layer, the source position can be obtained.

### 2.3. Improved Levenberg Marquardt algorithm of back propagation neural network

In order to speed up the learning process and reduce the training time, we should improve the traditional BP algorithm. Here, we use Levenberg Marquardt (LM) algorithm, a fast optimization algorithm that combines gradient descent method with Gauss-Newton method. It has not only the character of local convergence in Gauss-Newton method, but also the character of global convergence in gradient descent method [10]. As a result, it can be used to solve our reconstruction problem as an improvement of BP method.

Let $x^{\left(k\right)}$ be the weight vector of the kth iteration. The new vector $x^{\left(k+1\right)}$ can be written as

(2) $$x^{\left(k+1\right)}=x^{\left(k\right)}+\Delta x.$$

According to Newton method, we get

(3) $$\Delta x=-{\left[\nabla^{2}E(x)\right]}^{-1}\nabla E(x),$$

where $\nabla^{2}E(x)$ is called the Hessian matrix of the error criterion function E(x) and, ∇E(x) is called the gradient of E(x).

The error criterion function is $E\left(x\right)=\left(1/2\right)\sum_{i=1}^{N}e_{i}^{2}\left(x\right)$, where $e_{i}(x)$ is the error between the ith output and the ith target value

(4) $$\begin{array}{cc} \nabla E(x) & =J^{T}(x)e(x),\\ \nabla^{2}E(x) & =J^{T}(x)e(x)+J(x), \end{array}$$

where J(x) is called Jacobian matrix, as

(5) $$J(x)=|\begin{array}{l} \frac{\partial e_{1}(x)}{\partial x_{1}}\frac{\partial e_{1}(x)}{\partial x_{2}}\cdots \frac{\partial e_{1}(x)}{\partial x_{n}}\\ \frac{\partial e_{2}(x)}{\partial x_{1}}\frac{\partial e_{2}(x)}{\partial x_{2}}\cdots \frac{\partial e_{2}(x)}{\partial x_{n}}\\ ....\\ ....\\ \frac{\partial e_{N}(x)}{\partial x_{1}}\frac{\partial e_{N}(x)}{\partial x_{2}}\cdots \frac{\partial e_{N}(x)}{\partial x_{n}} \end{array}|.$$

For the Gauss-Newton method, we get

(6) $$\Delta x=-{\left[J^{T}(x)J(x)\right]}^{-1}J(x)e(x)$$

while LM is an improved Gauss-Newton method, the formation of which is

(7) $$\Delta x=-{\left[J^{T}(x)J(x)+\mu I\right]}^{-1}J(x)e(x),$$

where μ is the learning factor and I is the unit matrix. The basic steps of this method are as follows.

(i) Set the initial $x^{\left(k\right)}=x_{0}$, and take a large setting value of μ.

 (2) Calculate error criterion function $E(x^{\left(k\right)})$.

(3) If the function E is less than target or the number of training epochs reaches the fixed number, stop the training, else go on.

(4) Update $x^{\left(k\right)}$ to $x^{\left(k+1\right)}$ according to Equations (2) and (7).

(5) If $|E(x^{\left(k+1\right)})|\leq \left|E(x^{\left(k\right)})\right|$, then make $\mu^{\left(k+1\right)}=\alpha \mu^{\left(k\right)}$, where 0<α<1 else make $\mu^{\left(k+1\right)}=\beta \mu^{\left(k\right)}$ where β>1. Go back to ② .

## 3. SIMULATION

**Figure 2:**
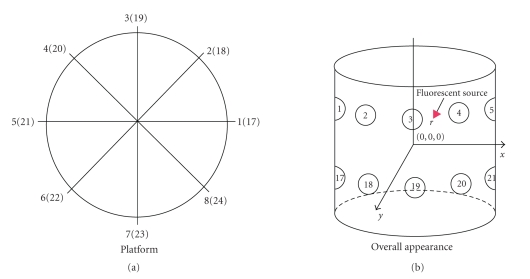
Cylinder phantom: platform and overall appearance.

Simulations were conducted in a homogeneous media as shown in Figure 2, in which the platform is shown on the left-hand side, and on the right-hand side is its overall appearance.

The cylinder, which the center is set at coordinate (0, 0, 0), has a height of 30 mm and 12 mm as the base radius. Each detector has a radius of 1 mm and the centers of the detectors are on the z-plane of z=6, and z=−6, respectively. The detectors in the two layers are numbered anticlockwisely (1–8, 17–24). Inside the cylinder, there is an absorptive sphere that has different absorption coefficient from the reference medium. To simplify the problem, the fluorescent source is assumed as a point source.

### 3.1. Simulation in 2D

In the first part of our simulation, we consider the estimation of source on the z-plane (z=6) and use the eight detectors in this plane to reconstruct the source position. We generate 10 groups of source positions randomly on the z-plane of z=6. For every source position, we get other 3 positions of its symmetrical position of x-axis, y-axis, and the center of the circle. For example, we randomly generate one coordination of (a, b, 6) and also get (a, -b, 6), (-a, b, 6), and (-a, -b, 6). Totally we get 40 data, each of which will be used later as the output of neural network during the process of training. Each time we use one of these 40 data to generate the information of eight detectors' (1–8) photon numbers through TBRS. Each of the 40 data we get by means of TBRS is used as the input of neural network that corresponds to the output. We generate other 30 coordinates on the same plane, as well as the information got by means of TBRS, as the testing samples. The 30 source positions for the test are selected every 36° on the circle of radius = 3, 6, and 9. When training is completed, we can use the testing samples to check out whether the network built by training works well in estimating the source position.

**Figure 3:**
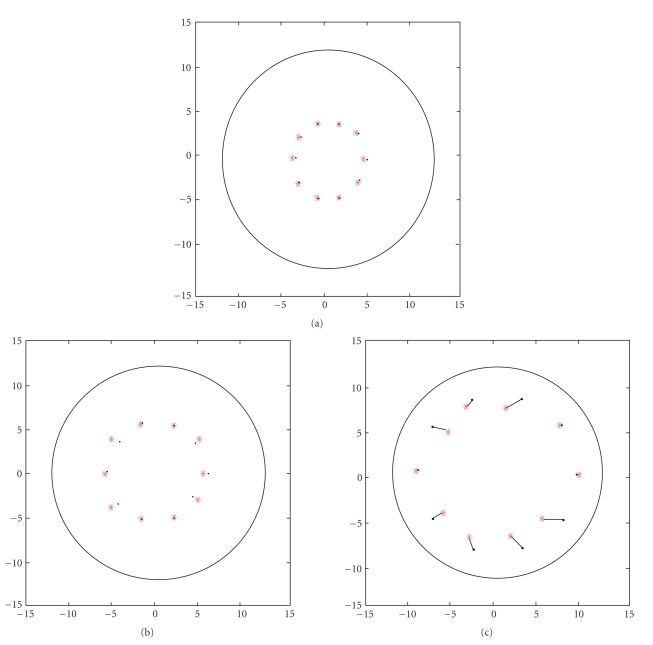
The comparison results between the estimated points and the actual ones.

When testing the new data, we define the term of “correct testing samples within the range of maximal allowable error.” When the distance between point coordinate calculated through the trained network and the actual one is smaller than 2.4 mm, we say the testing sample is correct. We also define the maximal allowable error as the ratio of the distance mentioned above to the diameter of the cylinder, 24 mm. Therefore, when the distance mentioned above is 2.4 mm, the maximal allowable error is 10%. Table 1 shows the result of improved LM algorithm of BP neural network simulation within the range of 10% allowable error. Figure 3 shows the distribution of the 30 samples.

### 3.2. Simulation in 3D

**Figure 4:**
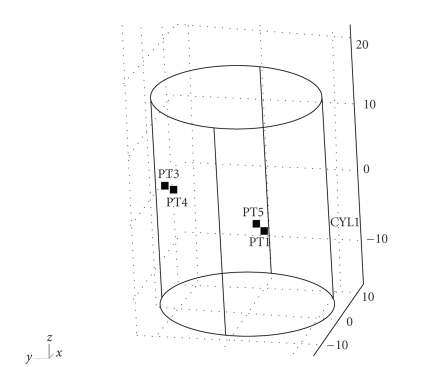
Two estimations of source position in the second simulation we select.

In the second part of our simulation, the coordinate (x,y,z) of source position is set randomly in a particular range. And we use all 16 detectors' photon numbers, since only 8 of them in z=6 or z=−6 may not determine the unique source position in 3D. In order to get higher accuracy of source position in the finite times of training, we should only select part of the cylinder instead of the whole region. When testing the new data that is also selected randomly in the same region as the training one, the maximal allowable error is defined the same as the first part of simulation. The result is shown in Table 2. We just select two results of estimation of source position to express our expected estimation more clearly (see Figure 4). It has proved to be a good result since it is hard to discriminate between the actual source position and the estimated one in 3-dimension.

## 4. DISCUSSION

The results have shown that backpropagation neural network can be implemented to position the source, though only in a particular region of the whole cylinder. The estimated source position can mostly be located in an acceptable range of error.

The first part of our reconstruction shows an especially good result due to our tactical selection of training samples. Not only does the result prove that backpropagation neural network can be implemented in the estimation of source position in 2-dimension, but it also presents a reliable selection of training samples, that is, to train one point as well as its other three symmetric points in the 2-dimension plane.

The result of our second part of simulation also shows a high accuracy of estimation, represented by its approximately 80% accuracy of the testing sample. The difference between the two correct rates of testing sample shows us that the nearer the region is to the detectors, the higher accuracy we get when testing, since the source point in Simulation 2 is closer to the detectors than it is in Simulation 1. From the result, we could see that it is possible that such region is apparently closer to some of the detectors that may make it easier for the neural network to learn in the process of training.

Actually, the shapes of training and testing region in the three simulations above are not as important as the dimension of it. In the second part of our simulation, we choose a cube region for training and testing just for the purpose that we can generate random numbers in our programing code more conveniently. We can also choose other shapes such as the cylinder, the sphere, and so forth. The dimension of the region is more pivotal, for a broad range of region would increase the difficulty in training. The 10×10×10 dimension in our simulation goes through many attempts in trying to make a balance between the low-convergence speed of training and the high accuracy of testing. On the other hand, we choose the whole circle plane as the region for training in our first part of simulation since it is much easier for network to learn in a lower dimension.

From the testing results, we can see that a high rate of accuracy was obtained after only forty training samples in the first part and twenty ones in the second part of our simulations. This demonstrates that the implementation of LM algorithm in BP neural network could make the convergence speed of training faster and the result of test more accurate. This method can be extended to solve the problem in which the source's shape is more complex and indefinite.

## Figures and Tables

**Table 1:** Estimation of source position on the z-plane of z=6.

Photon numbers	Number of training samples	Number of testing samples	Number of correct testing samples (maximal allowable error = 10%)	Maximal distance (mm)
10 000 000	40	30	30	2.18

**Table 2:** Estimation of source position in the particular region.

	Source point region (cm)	Number of training samples	Number of testing samples	Number of correct testing samples (maximal allowable error = 10%)	Maximal distance (mm)
Simulation1 in 3D	−5<x<5, −5<y<5, −5<z<5 randomly selected	20	40	31	7.7

Simulation2 in 3D	0<x<10, −5<y<5, −5<z<5 randomly selected	20	40	35	5.9
